# Emergent Homeostasis and Degeneracy From Multi‐Dimensional Attractors

**DOI:** 10.1002/bies.70116

**Published:** 2026-03-06

**Authors:** Kuheli Biswas, Hanna Salman, Naama Brenner

**Affiliations:** ^1^ Deptartment of Chemical Engineering and Network Biology Research Lab Technion ‐ Israel Institute of Technology Haifa Israel; ^2^ Deptartment of Physics and Astronomy Pittsburgh University Pittsburgh Pennsylvania USA

## Abstract

Biological systems maintain homeostasis, ensuring stability in the face of internal and external perturbations and counteracting stochastic noise. Traditionally, this is understood through the lens of control mechanisms designed to offset variations and maintain certain quantities near functionally desired set points. Here, we propose an alternative perspective to understand homeostasis: the *collective dynamics perspective*, in which homeostasis emerges spontaneously from high‐dimensional interactions, creating limiting manifolds in phase space. These multi‐dimensional attractor manifolds can constrain many components collectively, eliminating the need for explicit control of individual variables. The presence of null directions on a manifold allows for degenerate states that can add flexibility while preserving functionality. Using single‐cell growth and division homeostasis as a test case, we develop and support our perspective by models and meta‐analysis of numerous single‐cell datasets across organisms and conditions. Importantly, we do not reject the control‐theory perspective but rather suggest that control circuit models can be seen as low‐dimensional projections of a more complex, multi‐dimensional system.

## Graphical abstract

Biological systems maintain homeostasis—stability to perturbations. Control theory (right) describes this as designed regulatory circuits that maintain set points. We propose that homeostasis can emerge from collective dynamics that generate stable attracting manifolds (left). Perturbations displace trajectories, but dynamics restore them to the manifold. Degenerate directions allow variability without loss of function.

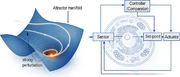

## 1 Introduction

Living systems maintain stability in many of their properties despite internal and external perturbations, a phenomenon known as homeostasis [1, 2, 3]. This property characterizes all scales of biological organization, from molecular circuits and cellular properties to organs and physiological function. When homeostasis fails, system dysfunction occurs, making it a subject of significant interest and extensive research over many years. The prevailing approach views homeostasis as the outcome of feedback control mechanisms that evolved to counteract molecular and environmental perturbations and keep certain quantities near functionally desired set‐point values. Control theory, with its mathematical and conceptual tradition, offers a natural framework for describing such processes and has been extensively applied for understanding biological homeostasis [4, 5, 6].

A challenge to this view is that living systems are inherently high‐dimensional, with countless interactions among their components, and maintain global function with statistical stability of multiple variables over time. Designing—or even conceptualizing—a set of controls that simultaneously regulate many specific target values is highly challenging. This raises a fundamental question: how is such global homeostasis achieved?

High‐dimensional dynamical systems can naturally give rise to **attractors** that collectively constrain multiple variables, reducing or even eliminating the need for dedicated feedback circuits that stabilize each of them. The attractor concept generalizes the familiar notion of a fixed point (a zero‐dimensional attractor) to higher‐dimensional structures, such as limit cycles or line attractors.

In this paper, we propose and develop the idea that biological homeostasis can emerge from the intrinsic collective dynamics of the system. In this perspective, stability is not imposed by explicit feedback targeting specific set points, but arises as an emergent property of the underlying dynamics. Attractors (or limiting manifolds) play a crucial role in these emergent dynamics; our discussion therefore centers on understanding the emergence, geometry, and properties of such manifolds.

Attractors and stable limiting manifolds can induce homeostasis by spontaneously constraining perturbations in multiple variables, as they attract high‐dimensional trajectories back toward them. An additional potential mechanism contributing to homeostasis can stem from degenerate directions of an attractor. On a smooth limiting manifold, trajectories may be free to evolve along any direction within the manifold while restricted from moving away from it. Biologically, this would imply that the system's functionality is insensitive, or degenerate, with respect to some variables, suggesting that they need not be regulated. Interestingly, degeneracy of this type has been independently observed in various biological contexts [7, 8], and we argue that it plays a role in achieving system‐level homeostasis.

We focus on the biological cell, the fundamental unit of life, as a model system to present and develop our perspective. Exploring homeostasis and variability in cellular growth and division, we demonstrate how a collective dynamic attractor, emerging from complex cellular interactions, enforces multi‐variable homeostasis. We characterize the geometric properties of this attractor, and identify two distinct types of variability: *temporal fluctuations within a single cell*, which are constrained by the attracting nature of the collective attractor; and *persistent variability among individuals*, which does not harm homeostasis, and may enhance adaptability while preserving functionality. Our conclusion is that, in proliferating cells, homeostasis can maintain stable growth and division, even while observable quantities such as cell size or protein content can vary significantly.

The collective dynamic perspective and its predictions, as well as the distinction between the two types of variability, are supported by a broad set of single‐cell measurements that align with these predictions.

## 2 Collective Dynamic Attractors of Growth and Division

Recent advances in single‐cell experimental techniques, allow the quantitative study of cellular growth dynamics with high statistical power and time span [9, 10]. Single cells display significant variability in all measurable properties such as shape and size, molecular content, growth rate, etc. This variability is observed even when their genomes are identical, and they are all under homogeneous environmental conditions. At the same time, the dynamics of proliferating cell populations maintain homeostasis of growth and division over multiple cycles. Moreover, this homeostasis entails balance between multiple cellular components. These properties have highlighted several questions: what mechanisms control growth and division homeostasis? How are individual cellular components coordinated and stabilized?

Here, we explore *collective dynamic attractors* in models of cellular growth and division, highlighting how they regulate temporal fluctuations and examining their underlying geometric structure. To this end, we focus on processes within a single cell, analyzing growth and division through the lens of dynamic attractors. As we will see, the dimensionality of the model plays a crucial role in determining the range of possible behaviors.

*Fixed points in one‐dimensional models*. At a coarse‐grained level, cell growth and division can be described using a discrete mapping from one cell cycle to the next. Recent studies often model this process by focusing on a single variable, such as cell size or protein content. Consider a cell starting with an initial size $V_{n}$ at cycle n. If it multiplies by a factor m over the cell cycle and divides by a factor f at the end of the cycle, the discrete map is given by:

(1) $$\begin{array}{c} V_{n+1}=f\cdot m\cdot V_{n}. \end{array}$$

As an example, many bacteria divide symmetrically and, on average, double their size over the cell cycle, with m=2,f=1/2. Theoretically, this mapping is sensitive to fluctuations in m and f: due to its multiplicative nature, such fluctuations accumulate over time and can lead to instability. In the absence of other negative correlations, even small fluctuations in these parameters would accumulate and disrupt homeostasis. Hence, in this one‐dimensional framework a division mechanism based on the doubling of its size is unstable.

In contrast, if we assume that the cell adds a fixed quantity of biomass or protein Δ and then divides in half, this process has a stable fixed point, allowing homeostasis to be maintained even in the presence of significant noise in Δ and f. This is the well‐studied “adder model” [11, 12], where the mapping is described by:

(2) $$V_{n+1}=f\cdot \left(V_{n}+\Delta \right).$$

It is easy to show that, with symmetric division f=1/2, this mapping has a stable fixed point $V^{*}=\Delta$. In the presence of noise both in f and in Δ, $V_{n}$ converges to a stable distribution around an average size ⟨V⟩=Δ, whose properties have been studied theoretically [11, 13]. This is an example of a coarse‐grained model of cell size control, where homeostasis is induced by implicit feedback arising from the attractor of the mapping. If cell division is triggered by the accumulation of some protein that adds a fixed value over the cycle, and assuming further a strong correlation between cell size and proteins ‐ namely, balanced biosynthesis ‐ one may motivate the adder model by known biological mechanisms [14].

This one‐dimensional level of description leaves several directions for refinement. First, continuous dynamics within the cell cycle are neglected; the mapping (2) could equally reflect exponential, linear, or any other form of accumulation between divisions. We argue below that the approximately exponential accumulation observed in many cases has significant implications to the underlying dynamics.

Second, the main empirical support for the adder control model is the independence of added size on initial size, across the range of initial sizes observed experimentally. However, while Δ was found empirically uncorrelated with initial cell size on average over large data sets, a more detailed analysis that examines individual lineages separately reveals a small negative correlation over time [15]. Additionally, compensation between sister cells was observed by tracking them for a full cell cycle after separation; the smaller sister exhibits, on average, a larger added volume [16, 17, 18], again in contradiction to the principle of fixed added size as a control trigger for division.

Finally, exponential accumulation characterizes not only cell size but also highly expressed proteins. The rate of this accumulation fluctuates across cycles [12, 15, 19, 20, 21, 22, 23], with strong correlations observed among various cellular components [15, 20, 23]. Even if variables grow independently with the same rate across the cycle, stability against accumulating fluctuations is not achieved, suggesting additional interactions or mechanisms [15, 24]. We shall see that a higher dimensional dynamic model can provide a more refined and quantitative characterization of multi‐variable homeostasis, while overcoming the instability problem that arises in the independent‐variable picture.

*Attractors in high‐dimensional models*. Balanced exponential growth can naturally emerge in a network of coupled chemical kinetics, as observed many years ago [25]. An arbitrary system of coupled linear reactions typically converges to exponential growth of all components at the same rate. This occurs because, in linear systems, the largest eigenvalue of the interaction matrix dominates as time progresses, forming an asymptotic dynamic attractor: as time goes by, all trajectories are attracted towards a manifold on which all components increase exponentially at the same rate. The manifold itself is an invariant of the dynamics: a system starting on this 1D structure will remain on it (such a manifold is not an attractor in the strict mathematical sense, since it is not a bounded structure). In systems with many variables and a randomly chosen interaction matrix, the presence of at least one positive eigenvalue is highly likely, making exponential growth the typical long‐term behavior [15, 26]. While this growth is universal, the specific rate λ (inverse of the largest eigenvalue) depends on the details of the interactions and the parameter values.

Recent theoretical work [26, 27] demonstrates that dynamic attractors of balanced exponential biosynthesis emerge also in a broader class of models with *non‐linearly* interacting components. This result, observed in several specific models [23, 28], was found to characterize a large class of kinetic reaction networks. While reaction kinetics are often expressed in terms of concentrations, the mathematical condition for this class of networks is simplest to formulate in absolute quantities. If X is a vector of molecular species copy number interacting within a volume composed of their linear combination $V=\Sigma \rho_{i}X_{i}$, then any system of interactions $\dot{X}=f\left(X\right)$ obeying the scalability condition:

(3) f(βX)=βf(X)

belongs to this class. Notably, all standard kinetic interactions ‐ linear and nonlinear ‐ are included (as can be seen by transforming between concentrations and copy numbers; for further details, see [26, 27] and Note S1). Thus, similar to the linear case, a nonlinear network of chemical reactions in a growing volume will typically exhibit long‐term dynamics where all components accumulate exponentially with the same rate ‐ balanced biosynthesis. The generality of this result presents a robust theoretical basis, which motivates the use of this class of models to understand cellular dynamics in a general framework.

In the balanced state, the ratios between any two components growing at rate α, remain fixed along the invariant manifold, ensuring coordinated growth: if $X_{1}$ and $X_{2}$ are two such components,

(4) $$\frac{X_{1}\left(t\right)}{X_{2}\left(t\right)}=\frac{X_{1}\left(0\right)e^{\alpha }t}{X_{2}\left(0\right)e^{\alpha }t}=\frac{X_{1}\left(0\right)}{X_{2}\left(0\right)}.$$

**FIGURE 1:**
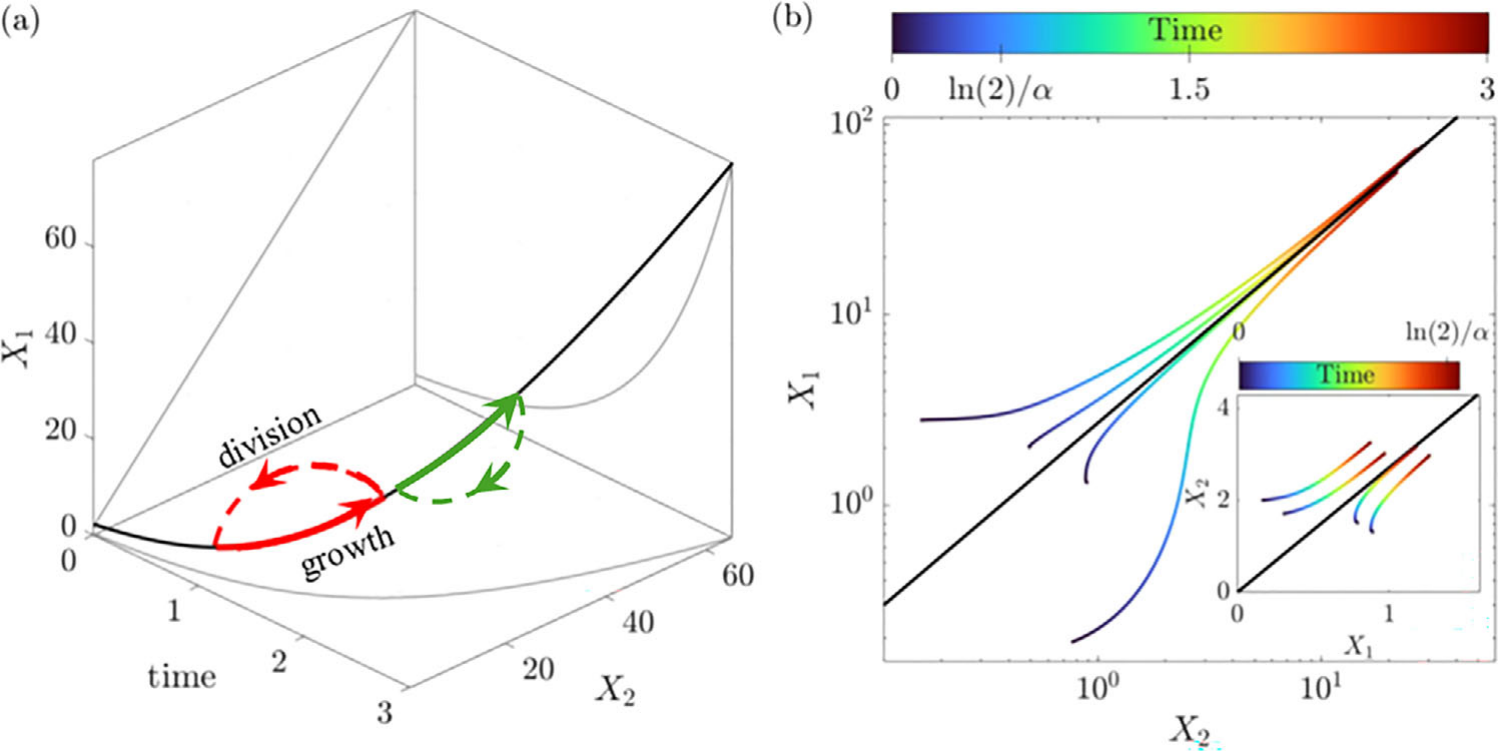
Curve of balanced exponential growth in high‐dimensional phase space of cellular dynamics. A model of three interacting components in a growing volume was simulated (details in Note S2). (a) Black line: the curve of balanced exponential growth in the space $\left(X_{1},X_{2},t\right)$. Gray lines: projections of the curve on the $\left(X_{1},t\right)$ and $\left(X_{2},t\right)$ planes, demonstrating exponential growth; projection on the $\left(X_{1},X_{2}\right)$ plane highlighting the fixed ratio between components. Red and green: cycles of growth and division. Starting from an initial condition on the curve, continuous growth proceeds to remain on it and symmetric division maps discontinuously to another point on the curve. (b) Starting from arbitrary initial conditions, dynamic trajectories are drawn closer to the curve of balanced exponential growth as time progresses. The doubling time ln(2)/α, is marked on the time color code; note the logarithmic axes, and the relatively long timescale for convergence. Inset: Trajectories with arbitrary initial conditions in linear scale and over the limited time interval 0 to ln(2)/α.

Cell size, a linear combination of its components, also increases exponentially with the same rate. As a result, molecular concentrations (i.e. the copy number of each component per cell volume) remain fixed on the manifold. Figure 1a shows the curve of balanced exponential growth for a specific model system [23, 26], as a black solid line in the space including two out of the three dynamic variables and time. The exponential growth of each component is depicted as grey solid lines on the projection planes of the component and time, $\left(X_{1},t\right)$ and $\left(X_{2},t\right)$. The projection on the two components $\left(X_{1},X_{2}\right)$ forms a straight line, representing the fixed ratio between $X_{1}$ and $X_{2}$ on the attractor.

Unlike a fixed point, the curve of balanced exponential growth is inherently dynamic. It is unstable in the sense that all variables increase exponentially at long times, but comprises a stable structure in phase space, continuously drawing nearby trajectories toward it; in this sense it is a generalization of a fixed point. Its stability is demonstrated by examining trajectories originating from various arbitrary initial conditions. Figure 1b illustrates these trajectories in the $\left(X_{1},X_{2}\right)$ plane (color coded for time), with the asymptotic attractor depicted by a black line. As time advances, the trajectories converge increasingly closer to the limiting curve. Note that the trajectories are depicted in logarithmic scales; the doubling time, a characteristic timescale of the dynamics, is marked in the color‐coded representation. The inset of Figure 1b presents the same trajectories on a linear scale, terminated at the doubling time.

Additional low‐dimensional projections provide intuition into the nature of the balanced exponential growth curve and its attractive nature (Note S2). One projection (Figure S1) shows the phase plane $\left(V,\dot{V}\right)$, demonstrating the global attraction of all trajectories to the straight line $\dot{V}=\alpha V$. The other (Figure S2) is a projection of the dynamics in a different coordinate system, of (n−1) component ratios and the size: $\left\{X_{i}/X_{1},V\right\}$, highlighting the fast convergence in the ratio directions. Previous work has shown that this type of dynamical system, projected on the (n−1)‐dimensional simplex of concentrations, exhibits a true fixed point and an ergodic measure [27].

Applying these theoretical insights to a biological cell, we note that continuous growth proceeds for a limited time, approximately doubling its constituents, before division occurs. For perfectly proportional division (symmetric or asymmetric), all components preserve their ratios and keep the system's trajectory on the attractor. This results in a hybrid continuous‐discrete cycle of growth and division, in which a cell can, in principle, persist infinitely [26]. Example cycles are illustrated in Figure 1a in red and green. During each cycle, continuous balanced exponential growth occurs along a finite segment (solid line) of the attractor, which extends to infinity (solid black line). At the discrete event of division, the system “jumps” back to its initial state (dashed line), ready to begin the next cycle. Throughout this process, all components remain finite over time, and all ratios remain fixed. This picture is robust with respect to the mechanism of division control [29]. We show in Note S3, that coupling to a well‐known dynamic model of division control [30] also retains these properties.

In reality, the growth and division of a single cell or lineage exhibit stochastic behavior over time. Division is not precise, the instantaneous exponential growth rate fluctuates, and the effective growth rate over an entire cell cycle varies across cycles. We next discuss the effects of fluctuations on the dynamics near the curve of balanced exponential growth, and the finite‐time growth and division cycles. We specifically identify two distinct types of variability, as described next.

### 2.1 Type‐1 Variability: Temporal Fluctuations Restrained by the Dynamic Attractor

Temporal fluctuations arise from many different sources and perturb the perfect growth‐division cycles described above. However, as we have seen, the attractive nature of the balanced exponential growth curve ensures that, following deviations, trajectories are pulled back toward it. This enables homeostasis across many variables simultaneously, mitigating temporal fluctuations without the need for their estimation or compensation individually, and without the need for a dedicated coordination mechanism between them. Evidence of this emergent homeostasis can be identified by analyzing the statistical properties of experimental single‐cell data. Below, we present three key predictions regarding these statistical properties and demonstrate their validation in experimental data.

The first prediction arises from deviations from perfectly symmetric cell division, which can distribute various cellular components differently among daughter cells. This stochastic nature of division randomly resets the starting points of cell cycles to different nearby locations in phase space. As a result, the smooth growth along the fixed‐ratio attractor is disrupted, temporarily perturbing the concerted time‐evolution of cellular variables and causing deviations from the attractor. This deviation leads to fluctuations in instantaneous growth rates across cycles at their initial parts. However, as the cell cycle progresses, trajectories gradually return to the attractor, and growth rates stabilize, converging nearer to the steady‐state growth rate characterizing the attractor. This would imply a decreasing variability in instantaneous growth rate across individual cell cycles.

**FIGURE 2:**
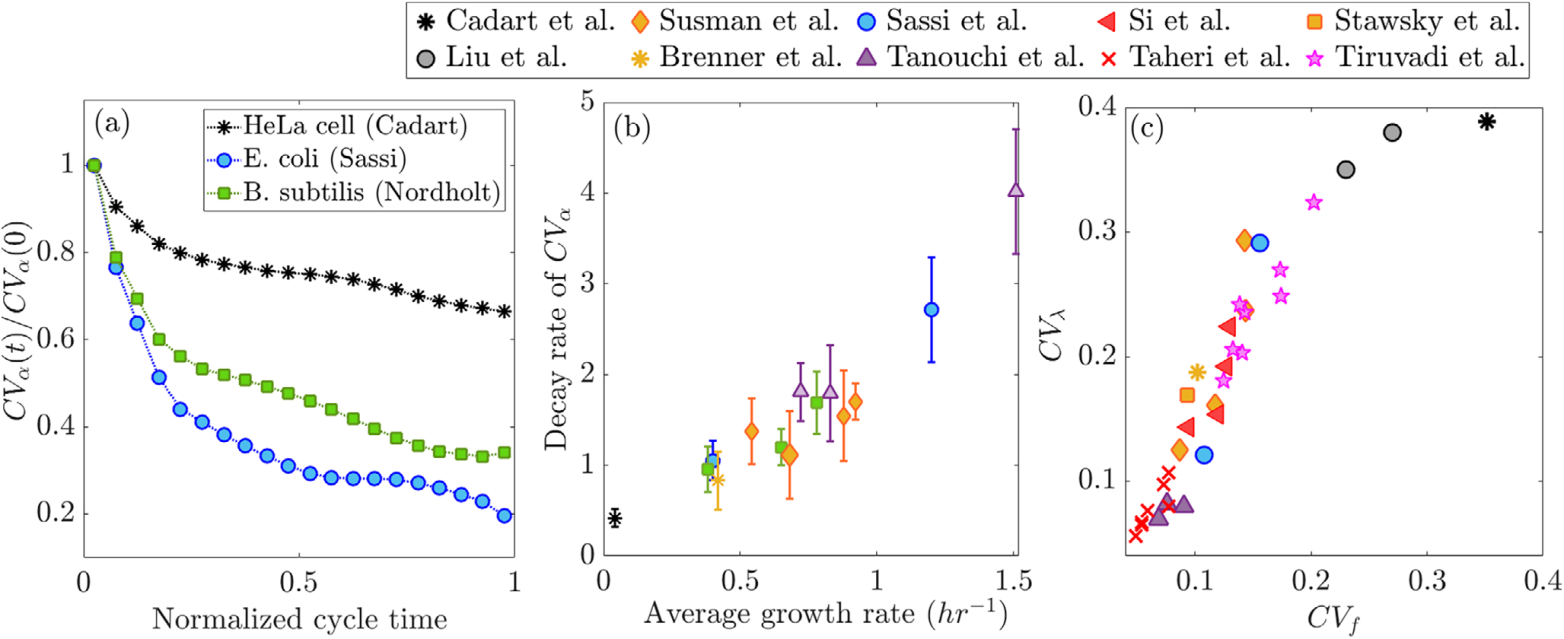
Experimental data support predictions of collective dynamic perspective on statistical properties of growth rate. (a) Decrease of CV (standard deviation over mean) in growth rate, over normalized cycle time, reflects the random initial conditions following noisy division, and the attraction of trajectories over time towards the curve of balanced exponential growth. Data from *E. coli* [23] and *B. subtilis* [21] bacteria and mammalian cells [31]. (b) The decay rate of $CV_{\alpha }$ is derived from an exponential fit to the curve in (a). It is a dimensionless quantity measuring the decay in fractions of the cell cycle. Each point represents one experiment that includes multiple lineages; the symbols are the average and the error bars ‐ the standard deviation over lineages. (c) According to the collective dynamic picture, a noisy division is a primary source of disruption to the curve of balanced exponential growth and thus a major driver of effective growth rate variability. The data were collected from various experiments, including bacterial and mammalian cells grown under diverse environmental conditions [12, 14, 15, 19, 20, 21, 22, 23, 31, 32, 33].

To test this prediction, we estimate the coefficient of variation ($CV_{\alpha }$), the ratio of the standard deviation to the mean, of the instantaneous growth rate across cell cycles as a function of cycle progression. Figure 2a presents the results for three different cell types, including both microbial and mammalian cells. The observed decrease in CV of the growth rate throughout the cycle provides experimental support of our theoretical expectation.

The second prediction derived from the collective dynamic perspective concerns the rate of return to the attractor following division, quantified by the rate of crease of $CV_{\alpha }$ in Figure 2a. In specific theoretical models, this rate can be computed and increases with the attractor growth rate. This result is intuitive, since faster growth leads to quicker progression along trajectories, thereby accelerating the return to the attractor.

Experimental validation of this prediction is presented in Figure 2b. The figure shows the decay rate, obtained by fitting an exponentially decreasing function to the trends observed in Figure 2a, plotted as a function of the average growth rate. Each point represents an independent experiment encompassing multiple cell lineages; the symbols indicate the mean, while the error bars denote the standard deviation across lineages. These data are drawn from seven different experimental labs and span a diverse range of single‐cell measurements, including bacterial and mammalian cells grown under various environmental conditions. They reveal a clear increasing relationship between the average growth rate and the rate of decrease in variability, supporting the second theoretical prediction.

The third prediction directly connects growth rate variability to division noise. Here, we examine the effective growth rate over the cell cycle, which is determined by fitting the accumulation dynamics over the entire cycle with an exponential function. In our dynamic framework, division noise perturbs the time‐evolution of cellular components, causing their trajectories to deviate from the precise asymptotic growth rate. Nonetheless, since the dynamic range over one cycle is relatively small (approximately a factor of two), a simple exponential function with an effective exponent still provides a good approximation to the measured time trace. Its fitted value, however, will fluctuate depending on the magnitude of the deviation from the attractor at division: larger division noise is expected to cause stronger disruptions, leading to greater deviations in the trajectory and, consequently, larger variability in the effective growth rate. Indeed, Figure 2c confirms this relationship across a broad range of experimental data, spanning multiple organisms and cell types, and covering a large dynamic range in both axes. Each point represents an individual experiment, where the CV for both division noise ($CV_{f}$) and growth rate ($CV_{\lambda }$) were computed across the entire ensemble of cell cycles.

The above analysis suggests that cell division is a major disruption to the dynamic attractor. This leads to the conclusion that preventing cell division should extend the relaxation to the attractor, allowing the trajectory to reach closer to the attractor and thereby reducing growth rate noise further as more time passes. In that case, filamentous bacterial cells that elongate without cytokinesis due to inhibited division can provide another prediction: these cells should exhibit less growth rate variability than normally dividing cells as they continue to grow without division. This is consistent with recent findings [34], which align well with our theoretical framework.

These validated predictions reinforce the concept of a multi‐dimensional dynamic attractor characterized by fixed asymptotic growth rate, concentrations, and ratios. In this framework, homeostasis of all cellular components emerges naturally from the attractor's stability to perturbations over time. Next, we explore additional properties of the balanced exponential growth manifold, particularly its role in persistent variability.

### 2.2 Type‐2 Variability: Degeneracy on the Curve of Balanced Exponential Growth

Careful examination of single‐cell lineage growth and division data has revealed the presence of not only temporal fluctuations but also persistent variability among individual cells [15, 32]. This second type of variability, which we refer to as “type‐2” or persistent variability, is often overlooked. It becomes evident when averaging lineage data over time, which reveals that despite averaging over many cycles, variability among averages persists. Growth rate and cell size, in particular, are highly sensitive and display significant variation in their time‐averaged value across different lineages tracked within the same experiment and even in the same trap [32]. Notably, persistent variability does not accumulate and thus does not disrupt homeostasis; instead, each lineage seems to maintain growth and division homeostasis with distinct averages for many generations. This inherent type of degeneracy is naturally explained by the collective dynamics, as we now show.

The curve of balanced exponential growth, combined with multiplicative cell division, introduces an intrinsic degeneracy with respect to absolute quantities. In Figure 1a, two growth and division cycles are depicted (red and green), and others can arise from different initial conditions along the attractor. Thus, while perpendicular deviations are constrained by the attracting nature of the balanced growth curve, a flat direction permits lineage‐specific variation; lineages can coexist around different segments of the curve, resulting in distinct time‐averaged absolute quantities such as cell size and protein content.

**FIGURE 3:**
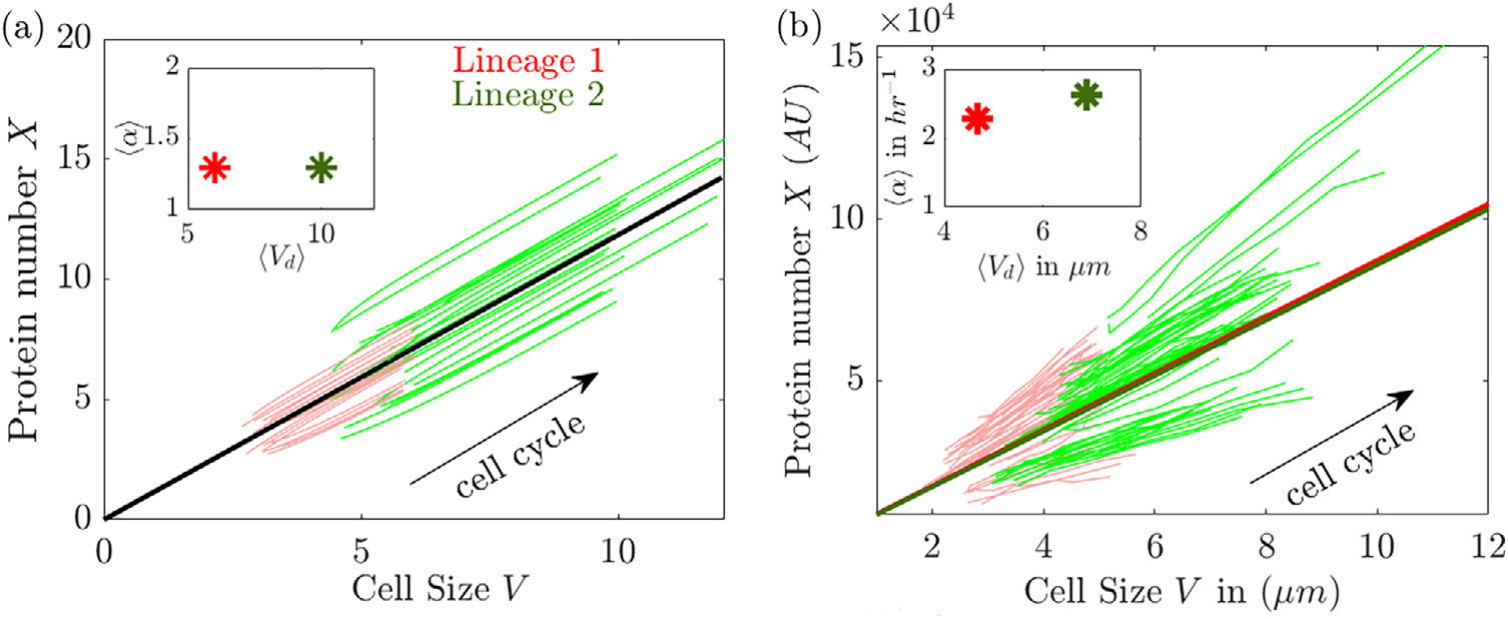
(a,b) Degeneracy in a single balanced growth curve: model and data. (a) Two lineages with different birth sizes and protein copy numbers (initial condition), obeying the same dynamics with the same parameters, maintain stable cycles of growth and division (thin lines, green and red) around different segments of the same curve of balanced exponential growth (dark thick straight line). Inset: Their average growth rate is the same, as determined by their common attractor, but their average cell size differs according to the region of the attractor. (b) Similar behavior of two lineages in the experiment. Experimental data are on wild‐type MG1655 *E. coli* at 30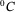 in LB medium (protein expressed from λ−pR promoter) [15].

To illustrate this effect, we simulated two lineages following the model dynamics, starting from different initial conditions and incorporating noisy division. In Figure 3a, the balanced growth curve is depicted by a heavy line, marking a fixed ratio between the two components (see projections in Figure 1). The two lineages (thin colored lines), each remain close to the attractor across multiple cycles but occupy distinct regions.

A comparable effect is observed in experimental data, as shown in Figure 3b. Here, two lineages measured within one experiment are presented in the same form as the simulation. The insets in Figure 3a,b highlight that the two lineages share a similar growth rate—suggesting the same attractor—yet maintain homeostasis around significantly different average cell sizes, corresponding to different degenerate regions on the attractor.

### 2.3 Parameter Variability in a Cell Population

We have seen that degeneracy is found in the absolute values of cellular components, and thus trajectories within a single dynamical system with fixed parameters can exhibit long‐term homeostasis around distinct regions of the same attractor. In addition, the system's parameters themselves can be modulated by internal perturbations, such as protein modifications, DNA configuration, and epigenetic or genetic regulatory modulation, as well as by external factors like temperature, chemical micro‐environment, or pH. Theory suggests that the qualitative properties of long‐term exponential growth are insensitive to specific parameter values [26, 27]. Thus, each distinct set of parameters gives rise to a separate stable curve of balanced growth having the same qualitative behavior discussed above.

**FIGURE 4:**
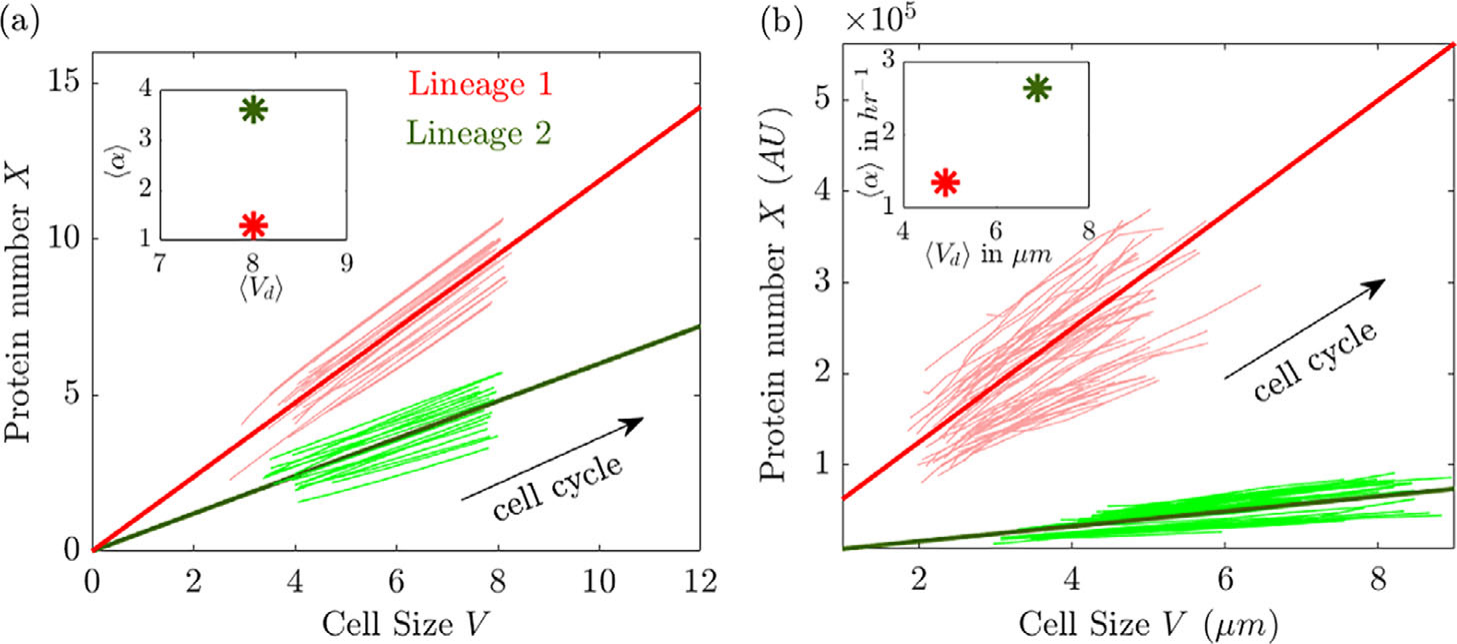
(a,b) Degeneracy across neighboring curves of balanced exponential growth: model and data. (a) Two lineages obeying the same dynamical system but with different parameters give rise to nearby attractors (dark green and red thick lines) in which qualitatively similar time evolution proceeds with stable cycles of growth and division. Each attractor defines a different protein‐to‐size ratio, reflected as a different slope. Insets: The two distinct attractors define distinct average growth rates, but due to the degeneracy of each attractor, the initial conditions can be chosen such that average cell size is the same. (b) Similar behavior of two lineages in experiment. Experimental data are on wild‐type MG1655 *E. coli* at 30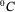 in LB medium (protein expressed from λ−pR promoter) [15].

For example, in microfluidic devices, the growth dynamics of cells in different channels may follow distinct attractors due to subtle environmental variations. Even within the same micro‐environment, epigenetic variability can shift a cell's trajectory to a slightly different attractor. Since each attractor maintains fixed component ratios, projections onto two‐component spaces appear as lines with different slopes. Figure 4a illustrates this effect in simulations, where two distinct attractors (thick lines) support separate multi‐generational trajectories (thin colored lines). The inset highlights that, in this case, the mean exponential growth rate differs between the two lineages, but due to each attractor's degeneracy, they can have similar average cell sizes. This behavior is mirrored in data from one experiment, as shown in Figure 4b, where two measured lineages exhibit parallel dynamics around two separate attractors, within the same experiment.

### 2.4 Homeostasis of the Biological Function in High‐Dimensional Parameters Space

The results and interpretation presented above suggest that cell size, absolute protein content, and even the precise exponential growth rate are not key variables determining the cell's functionality. Rather, these are “sloppy” variables that can exhibit persistent variability without compromising the biological function of growth and division homeostasis. This raises the question: what is the fundamental property that is maintained and regulated? Recent work suggests that the stability of the growth and division is captured by the total average change in cell size over multiple cycles of growth and division [32]. The compound variable encoding this property is the total fold change $r=fe^{\alpha \tau }$, where f is the division fraction and $e^{\alpha \tau }$ the fold growth (α ‐ cell size growth rate, τ ‐ cycle time). Homeostasis of growth and division is obtained when, on average over generations, $\bar{r}\approx 1$, regardless of the specific combination composing it, and regardless of the absolute cell size or content. With persistent variability among individual lineages, this can be maintained with diverse values of $\bar{\alpha },\;\bar{\tau }$ and $\bar{f}$ (all averaged over time within the lineage).

**FIGURE 5:**
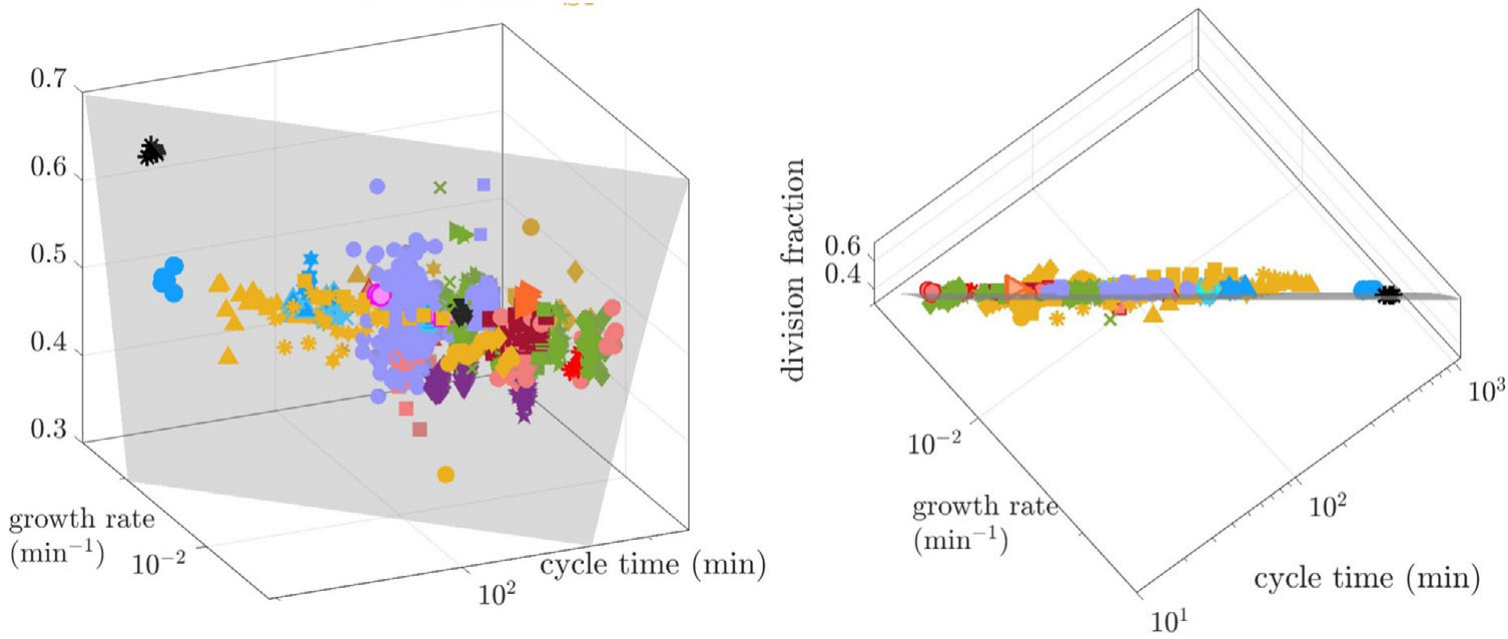
Lineage averages of cell cycle time ($\bar{\tau }$), growth rate ($\bar{\alpha }$), and division fraction ($\bar{f}$) reveal type‐2 variability. Each point represents a lineage, with its coordinates the three average values; shapes of the same color are different experimental conditions from the same lab (e.g., medium, temperature). All points lie close to the manifold $\bar{f}e^{\bar{\alpha }\bar{\tau }}=1$ (right; top view), indicating homeostasis of growth and division, but are widely scattered across it (left, front view) demonstrating type‐2 variability across lineages. Note the logarithmic axes of $\bar{\tau }$ and $\bar{\alpha }$, rendering the manifold flat. The data were collected from various experiments: [31] (black stars), [21] (pink symbols), [22] (purple symbol), [12] (green symbols), [23] (blue symbols), [19] (light blue symbols), [14] (ochre symbols), [20] (orange symbols), [15] (dark red symbols), [32] (maroon symbols). Details provided in the Note S4.

To test the generality of this principle, we analyzed a large dataset of single‐cell measurements, averaging each lineage's growth and division parameters over time and plotting these averages in the three‐dimensional space $\left(\bar{f},\bar{\alpha },\bar{\tau }\right)$. In Figure 5, each point represents a single lineage, where different colors denote different experiments. The manifold $\bar{f}e^{\bar{\alpha }\bar{\tau }}=1$ is depicted in gray. All data points adhere to the manifold, which approximates $\bar{r}=1$. Importantly, the data points are scattered along the individual coordinates—both within and across experiments, highlighting the compensation that persistent variability can provide: for instance, lineages with larger $\bar{\alpha }$ can maintain smaller $\bar{\tau }$ without compromising their long‐term homeostasis.

Note that all the data presented in Figure 5 is *time averaged*; therefore, type‐1 variability is eliminated, and the scatter represents exclusively type‐2 variability. The findings presented in this figure support a hierarchical picture, where individual growth parameters exhibit significant type‐2 variability across lineages, and homeostasis operates at a higher functional level—preserving the stability of the growth‐division process as a whole. Very likely, the scatter in the figure reflects both different attractors (i.e., distinct sets of model parameters) and degeneracy within a single attractor.

## 3 Discussion

The complexity of biological systems gives rise to emergent phenomena, where system‐level behavior cannot be fully understood or predicted by examining individual components separately. Accordingly, many fields in the life sciences have undergone a paradigm shift, driven by experimental advances that enable the simultaneous measurement of multiple variables. Notable examples include the ability to record the activity of hundreds to thousands of neurons over extended periods and to measure the expression of multiple genes within single cells. As these techniques continue to evolve, the structure of high‐dimensional dynamics is being unraveled, providing new insights into system‐wide phenomena. However, a fundamental challenge remains: how does high‐dimensional microscopic dynamics give rise to the organized behavior of a system? [35, 36].

Real‐time, simultaneous measurement of multiple variables (order 10) in single cells, while tracking growth and division, is rapidly advancing. Recent studies measuring both cell size and protein content have provided valuable insights into projections of the underlying high‐dimensional dynamics. Understanding these dynamics, along with their attractors and stability, is crucial for deciphering homeostasis in complex systems, such as the cell, where multiple components interact.

Here, we presented a collective dynamics perspective of cellular homeostasis developed by examining directly the high‐dimensional space of multiple interacting components, and supported it by experimental data. In this perspective, the target of homeostasis is stable growth and division for multiple cycles. The precise values of specific cellular variables, as well as the precise timing of division events, need not be strictly regulated to ensure such global homeostasis. Rather, it is ensured by the dynamic stability induced by an attracting manifold.

The theoretical framework is based on a recent identification of a broad class of dynamical systems, in which stable balanced exponential growth curve emerges asymptotically, leading to the coordinated exponential growth of all cellular components [26, 27]. In this class, the attractor emerges from the dynamics without fine‐tuning of the interactions or the number of components. The cell is approximated as a growing, structureless “bag of chemicals,” and the class includes all types of mass‐action kinetics. It is therefore expected to be highly relevant to bacterial cells; indeed, it is supported by a large set of data on different bacterial species in various conditions. In mammalian cells, it may be necessary to take into account structure and segregation inside the cell. Systematic deviations from balanced biosynthesis were studied in proliferating human cells and were found to be related to subcellular localization and organelle structure [37]. Nevertheless, we have included some eukaryotic cell data in our experimental tests as well, which follow qualitatively similar behavior to bacterial cells. An extensive comparison between bacterial and mammalian single‐cell data testing the current perspective is a topic for future work.

Studying the properties of the dynamic attractor allowed us to identify two distinct types of phenotypic variability with different relationships to homeostasis. First, temporal fluctuations, which can accumulate and endanger long‐term homeostasis: these are constrained by the phase space flow, which draws the trajectories back to a stable structure in this space.

Importantly, this structure is a collective manifold defined by relationships among multiple variables—a “curve of balanced growth” [26]. Imperfect cell division plays a key role as a disruption that perturbs the smooth coordinated propagation on the attractor, a perturbation orthogonal to the curve, that crucially affects the statistical properties measured in single‐cell experiments. Following the disruption caused by division events, regardless of the mechanism that controls them, all variables are simultaneously drawn back over the next cycle. This effect suggests that the collective dynamic perspective obviates the search for dedicated regulatory circuits that separately detect deviations in specific variables, compare them to set points, and actively correct them. Instead, it highlights global properties, which inherently stabilize a large number of system components simultaneously.

The second type of variability we identified is persistent individuality, which also arises from properties of the attractor. In previous work, ergodic theory was applied to the projected space of concentrations, where the attractor becomes a fixed point [27]. However, in the physical space of absolute quantities, the dynamic attractor supports multiple layers of biological degeneracy. First, for a fixed set of model parameters, the emergent (unique) attractor has a flat direction that allows for many solutions with varying properties, such as different average cell sizes, while maintaining stable component ratios and concentrations. Second, for small changes in cell physiology or local environment that would result in slight variation of model parameters, neighboring attractors emerge, each supporting the high‐level function of stable growth and division but with distinct physiological parameters, such as average growth rate. Note that these models, though high‐dimensional, are still coarse‐grained and include effective parameters that depend on hidden variables. This increases the chance of observing variability in effective parameters under nominally uniform experimental conditions.

These intrinsic degeneracies introduce persistent variability that distinguishes individual lineages without disrupting homeostasis. Unlike temporal fluctuations, such variability does not accumulate over time. On the contrary, it may even support homeostasis by allowing the system to settle into different attractor regions where stability is maintained. Similar forms of degeneracy have been discussed in other biological contexts [7, 8, 35]. Cells can leverage this degeneracy to preserve key functional ratios and concentrations that ensure proper replicative homeostasis while allowing variability in absolute quantities like protein content or cell size. Such degrees of freedom can enhance cellular adaptability to various environments and conditions by defining “null directions” along which the system can easily drift while maintaining functionality, as has been observed, for example, in high‐dimensional neural dynamics during motor learning [38]. Our recent work has identified that, among different phenotypic variables, some exhibit larger persistent variability than others; this could correspond to a “sloppy system” structure [39], defining a hierarchy of significance to function, where the least significant variable are allowed to vary among individual cells while co‐variation among them marks the more functionally relevant directions [32]. Understanding these analogies is a topic for future theoretical and experimental work.

While our perspective emphasizes emergent homeostasis, we do not propose a strict dichotomy between control theory and dynamical systems theory. Control systems are, in fact, a subset of dynamical systems, and the two frameworks are deeply interconnected. For instance, feedback loops regulating cell size or protein content can be interpreted as coarse‐grained approximations of the underlying high‐dimensional system. Alternatively, a simple control circuit might represent a low‐dimensional projection of a more complex dynamical system. It could also represent a separate module that is weakly connected to others [40]. A hallmark of complex biological systems is that they can be understood from multiple complementary perspectives [41]. Our goal is not to dismiss control‐theory approaches, but rather to complement them with a dynamical systems view of homeostasis, where stability emerges from the system's inherent structure rather than from explicit control mechanisms.

## Author Contributions

All authors contributed to carrying out the research and writing the paper.

## Funding

This research was partly funded by the United States‐Israel Binational Science Foundation Grant 2024215 (HS and NB), and the National Science Foundation Grant DMS‐2245816 (HS). K. B. is the recipient of the Lady Davis Post‐Doctoral Fellowship at the Technion.

## Conflicts of Interest

The authors declare no conflicts of interest.

## Supporting information


**Supporting File**: bies70116‐sup‐0001‐SuppMat.pdf.

## Data Availability

Data sharing is not applicable to this article, as no datasets were generated during the current study.
