# Supplementary material for: Emergent Homeostasis and Degeneracy From Multi‐Dimensional Attractors
Source: Bioessays. 2026 Mar 6;48(3):e70116. doi: 10.1002/bies.70116 (PMC12966630; doi:10.1002/bies.70116)
Supplement: Supplementary file 1 — Supporting File: bies70116‐sup‐0001‐SuppMat.pdf. [file BIES-48-e70116-s001.pdf]

Emergent Homeostasis and Degeneracy from Multi-Dimensional Attractors

SUPPLEMENTARY NOTES

Supplementary Note 1: General Model Formulation

Recent theoretical studies have shown that exponential growth with a single rate emerges in a broad class of models describing systems of non-linearly interacting components, such as chemical reaction networks (1, 2). For completeness we summarize here some of the results. Imagine the cell as a container of volume $V(t)$ containing n components, with copy numbers $X_i,\ i = 1, 2...n$. Dynamics of the ith component is given by

$$\dot{X}_i = F_i(\mathbf{X}), \tag{1}$$

where F_i are nonlinear functions, representing the system's chemical reactions. The cell size is a linear combination of its components, $V(\mathbf{X}) = \sum_{i=1}^{n} \rho_i X_i$. In such a system, one finds the emergence of an asymptotic *curve of balanced exponential growth*, where at long times all components increase exponentially with the same rate α^*:

$$X_i(t) = X_i(0)e^{\alpha^* t}, \quad i = 1...n. \tag{2}$$

This happens under the following scalability (or homogenity) condition on the functions $F_i(\mathbf{X})$:

$$F_i(\beta\mathbf{X}) = \beta F_i(\mathbf{X}) \quad \text{for any } \beta > 0. \tag{3}$$

The condition is obeyed by functions that describe mass-action kinetics of various order, and thus is very relevant to models of cellular interactions (1, 2). Consider two components X_i, X_j of the trajectory on the curve of balanced growth: if their ratio at time $t = 0$ is given, then that ratio is maintained for all times

$$\frac{X_i(t)}{X_j(t)} = \frac{X_i(0)e^{\alpha^* t}}{X_j(0)e^{\alpha^* t}} = \frac{X_i(0)}{X_j(0)}. \tag{4}$$

Additionally, the concentrations $Y_i = X_i/V = Y_i/\sum_{k=1}^{n} \rho_k X_k$ are also conserved for all i. To analyze this dynamical system in the space of concentrations instead of copy numbers, we write Eq. (1) as:

$$\dot{Y}_i = \frac{1}{V} F_i(V\mathbf{Y}) - \alpha(t)\, Y_i, \tag{5}$$

which includes also the effect of dilution by expanding volume, and where the volume instantaneous growth rate is defined as

$$\alpha(t) = \frac{\dot{V}}{V} = \sum_{i=1}^{n} \rho_i F_i(\mathbf{Y(t)}). \tag{6}$$

Supplementary Note 2: Explicit calculations for a special-case model

We consider a concrete example of a non-linear interaction network of three components: amino acids, metabolic enzymes, and ribosomes with respective population size X_{aa}, X_e, and X_r (1) :

$$\dot{X}_{aa} = k_1 X_e - (k_2 + k_3) \frac{X_{aa} X_r}{V} \tag{7a}$$
$$\dot{X}_e = k_2 \frac{X_{aa} X_r}{V} - \gamma_e X_e \tag{7b}$$
$$\dot{X}_r = k_3 \frac{X_{aa} X_r}{V} - \gamma_r X_r \tag{7c}$$
$$V(t) = \rho_{aa} X_{aa} + \rho_e X_e + \rho_r X_r. \tag{7d}$$

In this reaction network, amino acids X_{aa} are produced by the catalytic action of the metabolic enzymes X_e from external food molecules at a rate k_1. Metabolic and ribosomal proteins are synthesized from the amino acids in reactions catalyzed by ribosomes at rates k_2 and k_3, respectively, and are degraded with respective rates γ_e, γ_r. It is easily verified that the model obeys the scalability condition, and therefore exhibits an exponential attractor. The volume rate of change is computed explicitly for this system as

$$\begin{aligned} \dot{V}(t) &= \rho_{aa} \dot{X}_{aa} + \rho_e \dot{X}_e + \rho_r \dot{X}_r \\ &= \rho_{aa} \left(k_1 X_e - (k_2 + k_3) \frac{X_{aa} X_r}{V} \right) \\ &+ \rho_e \left(k_2 \frac{X_{aa} X_r}{V} - \gamma_e X_e \right) + \rho_r \left(k_3 \frac{X_{aa} X_r}{V} - \gamma_r X_r \right) \end{aligned} \tag{8}$$

and the instantaneous specific growth rate (iGR) is a function of the concentrations,

$$\alpha = \frac{\dot{V}}{V} = \rho_{aa}(k_1 Y_e - (k_2 + k_3) Y_{aa} Y_r) + \rho_e(k_2 Y_{aa} Y_r - \gamma_e Y_e) + \rho_r(k_3 Y_{aa} Y_r - \gamma_r Y_r). \tag{9}$$

The dynamic equations for the concentrations in this case are

$$\dot{Y}_{aa} = k_1 Y_e - (k_2 + k_3) Y_{aa} Y_r - \alpha(\mathbf{Y}) Y_{aa} \tag{10a}$$
$$\dot{Y}_e = k_2 Y_{aa} Y_r - \gamma_e Y_e - \alpha(\mathbf{Y}) Y_e, \tag{10b}$$
$$\dot{Y}_r = k_3 Y_{aa} Y_r - \gamma_r Y_r - \alpha(\mathbf{Y}) Y_r, \tag{10c}$$

with a unique fixed point found by setting $\dot{Y}_i = 0$

$$Y^*_{aa} = \frac{\alpha^* + \gamma_r}{k_3}, \tag{11}$$
$$Y^*_r = \frac{\alpha^*(\alpha^* + \gamma_e)}{k_1 k_2 - (k_2 + k_3)(\alpha^* + \gamma_e)}, \tag{12}$$
$$Y^*_e = Y^*_r \frac{k_2(\alpha^* + \gamma_r)}{k_3(\alpha^* + \gamma_e)}, \tag{13}$$

and the explicit expression for the growth rate at the fixed point

$$\alpha^* = k_1 \rho_{aa} Y^*_e + (k_2 \rho_e + k_3 \rho_r - (k_2 + k_3)\rho_{aa}) Y^*_{aa} Y^*_r - \rho_e \gamma_e Y^*_e - \rho_r \gamma_r Y^*_r. \tag{14}$$

We use this special case model to illustrate geometrically of the curve of balanced exponential growth on projections of the dynamics. Fig. 1 shows the $(V, \dot{V})$ phase plane with trajectories

starting at different points in the plane, demonstrating their attraction to the curve of balanced exponential growth, the diagonal with slop α^* (red dashed line). A different viewpoint can be seen by separating the dynamics of the ratios $X_{aa}/X_e,\ X_r/X_e$, which form an autonomous sub-system, from the dynamics of the volume V. Fig. 2 illustrates dynamic trajectories in this projected space, demonstrating how the ratios converge rapidly to their limiting value on the manifold of balanced exponential growth, and tend to grow exponentially as they approach it.

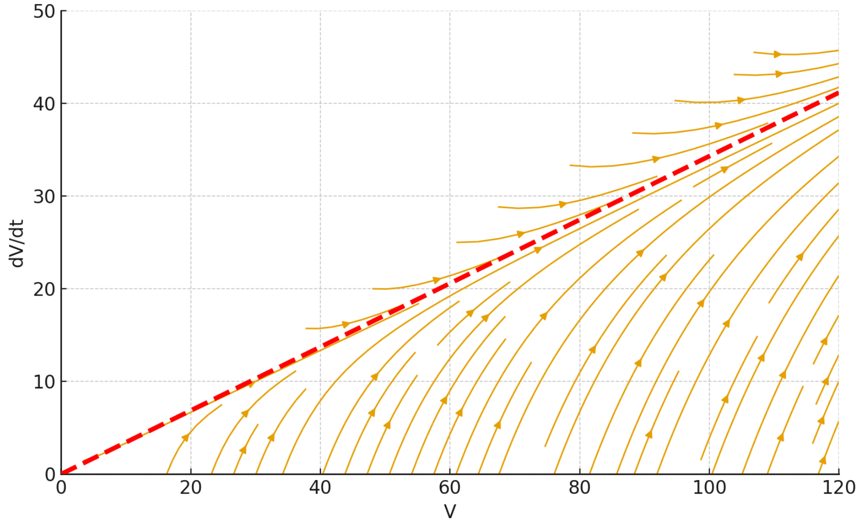

FIG. 1. **Dynamics in cell volume phase plane.** Trajectories of the model 7 in the projection plane $(V, \dot{V})$. The attracting curve of balanced exponential growth - on which $\dot{V} = \alpha^* V$ - is depicted as a dashed red line. Dynamic trajectories starting from different points in the plane are attracted to this curve and their direction tends to align with it as time progresses.

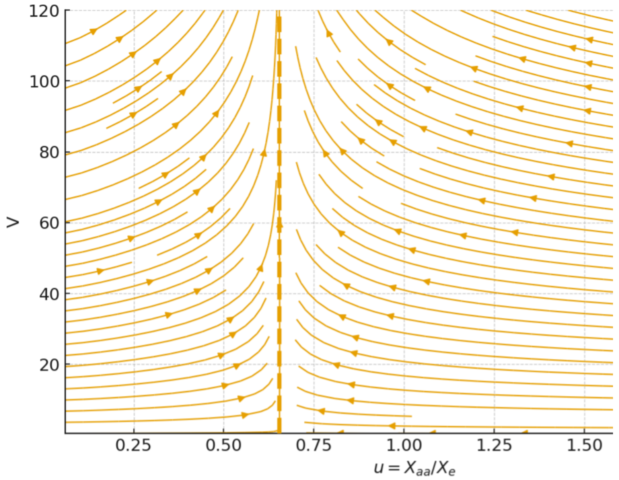

FIG. 2. **Ratio and Volume projection.** One of the two ratios (x-axis) and cell volume (y-axis) provide a different projection on the dynamics of the model 7. The limiting ratio defines the curve of balanced growth, and trajectories are attracted to this ratio while also aligning with the curve of balance growth on which V increases (vertical line in this projection).

Supplementary Note 3: Dynamic cell-cycle control model for division

Here we demonstrate insensitivity of our results to details of cell division. We couple the special-case model (Eq. 7) to a classic minimal regulatory circuit for mitotic control - a two-variable model for the dynamics of cyclin/CDK dimers (c) and active Cdh1/APC complexes (a), introduced by Tyson and Novák (3):

$$\dot{c} = k_1 - k_2 c - k_3 ac, \tag{15a}$$

$$\dot{a} = \frac{j_1(1-a)}{j_2 + 1 - a} - V(t)\,\frac{j_3 ac}{j_4 + a}. \tag{15b}$$

Concentrations are specified in the instantaneous cell volume $V(t)$, and k_i, j_i are kinetic parameters. The two species are mutual inhibitors: a promotes degradation of cyclin thereby reducing c, while c inhibits a through phosphorylation. By transforming to absolute copy number, it can be checked that this system obey the scalability property in all terms except for the one containing the cell volume, which was added to the model as an "external" parameter originally.

The cell volume $V(t)$ drives a bifurcation in the (a, c) dynamics as it increases throughout the cell cycle. When the volume is small, the system exhibits three fixed points, corresponding to a bistable switch with high/low concentrations of a and c respectively. As V increases, one of the nullcline is shifted and two fixed points are annihilated through a saddle-node bifurcation, leaving a single stable fixed point (see Fig. 3(a)). In turn, if a transitions from a high-activity state ($a \approx 1$) at small volumes to a low-activity state ($a \approx 0$) once V exceeds a critical threshold. This transition is interpreted as the cell-cycle commitment event that triggers division (3). At division, a is reset back to 1, and the cell volume is reduced by a factor 1/2.

To couple this cell-cycle module to our growth model, we take the volume to be determined self-consistently by the abundance of the coarse-grained components,

$$V(t) = \rho_{aa} X_{aa} + \rho_e X_e + \rho_r X_r, \tag{16}$$

where the variables $\mathbf{X} = (X_{aa}, X_e, X_r)$ evolve according to Eq. 7. Coupling the dynamics of these two additional components with the original model 7, we find that the system still exhibits balanced growth: Fig. 3(b) illustrates the curve of balanced exponential growth in the (X_1, X_2) projection plane (black curve); its attractive properties are maintained, as demonstrated by trajectories starting nearby and converging toward it (colored lines, encoding time).

Sample plots of individual components along time are presented in Fig. 4(b); note that coordinated exponential growth persists despite one term in the equation not obeying the scalability condition. Also shown is the concentration a (Fig. 4(a)). As cell size increases, this concentration decreases until it triggers division when crossing a threshold. Finally, comparing to the experimental results presented in the main text, we find that under this division control, CV_λ (CV of effective growth rate) depends strongly on CV_f (CV of the division noise) (Fig. 4(c)). This result is also consistent with the behavior observed for the simpler size-dependent division rules used in (4).

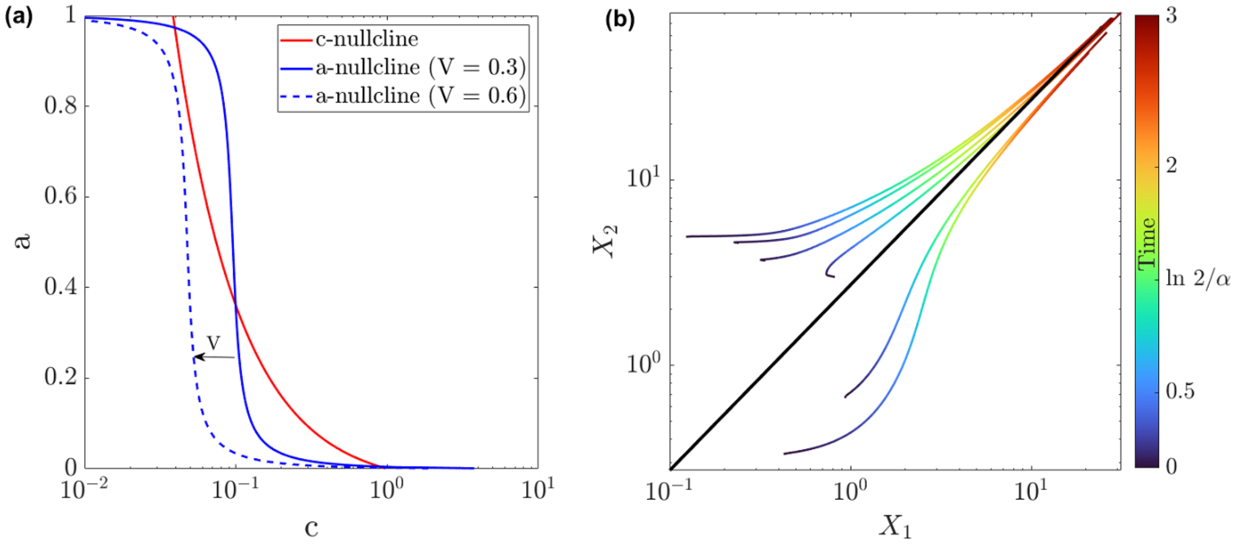

FIG. 3. **Tyson-Novák cell-cycle control model.** **(a)** Nullclines in the (a, c) plane. The cell-cycle regulator is coupled to the growth model via the volume $V(t)$; as cell size V increases, one of the nullclines shifts and induces a saddle-node bifurcation. **(b)** Curve of balanced exponential growth in the total system coupling the original model 7 with 15. If there is no division event in finite time intervals, then trajectories converge to the balanced exponential growth curve over time, starting from arbitrary initial conditions. The doubling time $\ln(2)/\alpha$ is marked on the time color code.

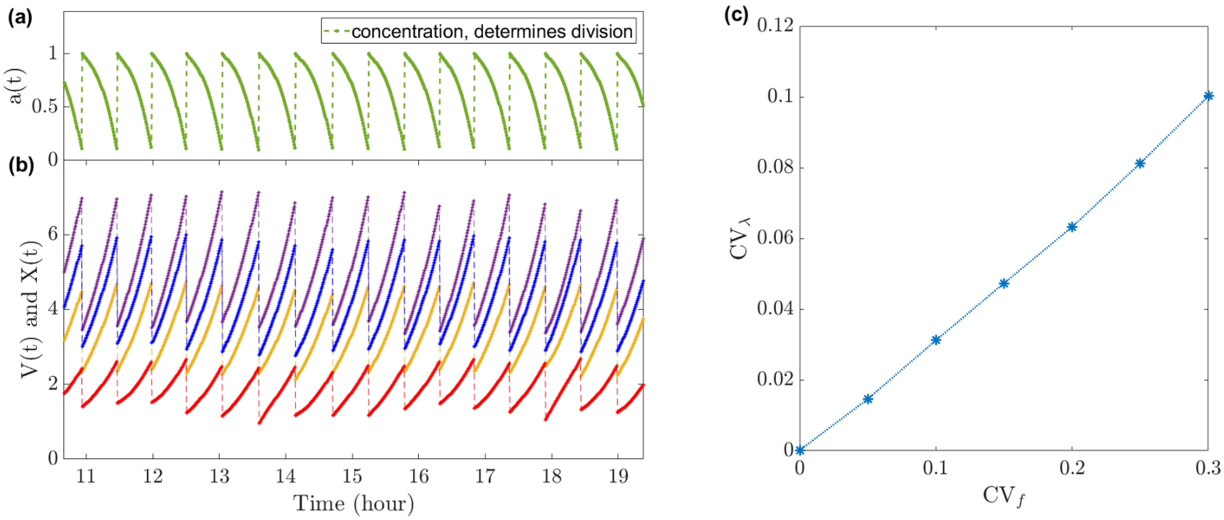

FIG. 4. **Balanced growth in the presence of a cell-cycle control module triggering division.** **(a, b)** The cell-cycle regulator is coupled to the growth model via the volume $V(t)$. Division is triggered when the regulatory variable, concentration of APC/C^{Cdh1}, $a(t)$, crosses a threshold from its high-activity state to its low-activity state as $V(t)$ increases. The system maintains balanced exponential growth, as the volume and the number of other proteins, $\mathbf{X}(t)$, grow exponentially at the same rate. **(c)** The coefficient of variation (CV) of the effective growth rate (eGR) is strongly dependent on the CV of the division noise, a property qualitatively shared by the data as well.

Supplementary Note 4: Details of experimental data analyzed

Strain	growth medium	#cell cycles	Temp.	mean birth size (μm)	mean growth rate (min^{-1})	References
E. coli in NCM3722 background	glycerol	1.12×10^4	37 ^0C	2.07	0.0189	(5)
	sorbitol	7439		2.26	0.0187	
	glucose	1.25×10^4		2.1	0.0270	
	glucose + 6 a.a.	1.68×10^4		2.35	0.0330	
	glucose + 12 a.a.	1.33×10^4		2.87	0.0372	
	Synthetic rich	1.09×10^4		3.33	0.0433	
	Tryptic soy broth	1.11×10^4		3.98	0.0569	
E. coli in NCM3722 background	arginine	1701	37 ^0C	1.48	0.0067	(6)
	glucose + 12 a.a.	1464		2.82	0.0234	
	glucose	1432		1.89	0.0144	
E. coli in MG1655 background	M9 acetate	1554		1.88	0.0034	
	glucose	1807		2.51	0.0131	
	glycerol + 11 a.a.	1491		2.67	0.0109	
E. coli in MC4100 background	LB	4550	25 ^0C	1.96	0.0121	(7)
		3780	27 ^0C	1.64	0.0149	
		1.12×10^4	37 ^0C	1.98	0.0253	
B. subtilis	arabinose	1.58×10^4	37 ^0C	2.07	0.0062	(8)
	glucose	1.25×10^4		2.477	0.0108	
	glucose + 4 a.a.	2887		4.105	0.0133	
E. coli in MG1655 background	poor	9.56×10^4	30 ^0C	1.93	0.0086	(9)
	rich (RDM)	4.64×10^4		3.28	0.0153	
E. coli in STK13 background	alanine-TrEl	215	28 ^0C	1.75	0.0033	(10)
	glucose-Cas	409		2.01	0.0099	
	mannose	401		1.5	0.0035	
	glucose	344		1.8	0.0061	
	glycerol-Cas	420		1.9	0.0074	
	glycerol-TrEl	423		1.61	0.0046	
	glycerol	302		1.7	0.0042	
E. coli in JM85 background	glucose-Cas	406		2.1	0.0092	
	glycerol	388		2.01	0.0044	
Wild type MG1655 *E. coli* under the control of *lac* promoter	M9 minimal	381	30 ^0C	2.31	0.0229	(11)
Wild type MG1655 *E. coli*	LB	4096	30 ^0C	2.65	0.0357	(12)
Wild type MG1655 *E. coli* under the control of λ promoter	M9 minimal	1794		1.35	0.0509	
	LB	3551		2.78	0.0252	
Wild type MG1655 *E. coli* under the control of *lac* promoter	M9 minimal	562		2.43	0.0233	
Wild type MG1655 *E. coli* under the control of both λ & *lac* promoter	LB	188		1.21	0.0257	

[1] P. P. Pandey, H. Singh, and S. Jain, "Exponential trajectories, cell size fluctuations, and the adder property in bacteria follow from simple chemical dynamics and division control," *Physical Review E*, vol. 101, no. 6, p. 062406, 2020.

[2] W.-H. Lin, E. Kussell, L.-S. Young, and C. Jacobs-Wagner, "Origin of exponential growth in nonlinear reaction networks," *Proceedings of the National Academy of Sciences*, vol. 117, no. 45, pp. 27795–27804, 2020.

[3] J. J. Tyson and B. Novák, "Cell cycle controls," in *Computational Cell Biology* (C. P. Fall, E. S. Marland, J. M. Wagner, and J. J. Tyson, eds.), pp. 261–305, New York, NY: Springer, 2002.

[4] K. Biswas, A. E. Sanderson, H. Salman, and N. Brenner, "Single-cell growth rate variability in balanced exponential growth," *bioRxiv*, 2024.

[5] S. Taheri-Araghi, S. Bradde, J. T. Sauls, N. S. Hill, P. A. Levin, J. Paulsson, M. Vergassola, and S. Jun, "Cell-size control and homeostasis in bacteria," *Current biology*, vol. 25, no. 3, pp. 385–391, 2015.

[6] F. Si, G. Le Treut, J. T. Sauls, S. Vadia, P. A. Levin, and S. Jun, "Mechanistic origin of cell-size control and homeostasis in bacteria," *Current Biology*, vol. 29, no. 11, pp. 1760–1770, 2019.

[7] Y. Tanouchi, A. Pai, H. Park, S. Huang, R. Stamatov, N. E. Buchler, and L. You, "A noisy linear map underlies oscillations in cell size and gene expression in bacteria," *Nature*, vol. 523, no. 7560, pp. 357–360, 2015.

[8] N. Nordholt, J. H. van Heerden, and F. J. Bruggeman, "Biphasic cell-size and growth-rate homeostasis by single bacillus subtilis cells," *Current Biology*, vol. 30, no. 12, pp. 2238–2247, 2020.

[9] A. S. Sassi, M. Garcia-Alcala, M. Aldana, and Y. Tu, "Protein Concentration Fluctuations in the High Expression Regime: Taylor's Law and Its Mechanistic Origin," *Physical review X*, vol. 12, no. 1, p. 011051, 2022.

[10] S. Tiruvadi-Krishnan, J. Männik, P. Kar, J. Lin, A. Amir, and J. Männik, "Coupling between DNA replication, segregation, and the onset of constriction in Escherichia coli," *Cell reports*, vol. 38, no. 12, p. 110539, 2022.

[11] N. Brenner, E. Braun, A. Yoney, L. Susman, J. Rotella, and H. Salman, "Single-cell protein dynamics reproduce universal fluctuations in cell populations," *The European Physical Journal E*, vol. 38, no. 9, pp. 1–9, 2015.

[12] L. Susman, M. Kohram, H. Vashistha, J. T. Nechleba, H. Salman, and N. Brenner, "Individuality and slow dynamics in bacterial growth homeostasis," *Proceedings of the National Academy of Sciences*, vol. 115, no. 25, pp. E5679–E5687, 2018.
